# Clinical and imaging features of surgically treated low lumbar osteoporotic vertebral collapse in patients with Parkinson’s disease

**DOI:** 10.1038/s41598-021-93798-1

**Published:** 2021-07-09

**Authors:** Hideaki Nakajima, Arisa Kubota, Shuji Watanabe, Kazuya Honjoh, Akihiko Matsumine

**Affiliations:** Department of Orthopaedics and Rehabilitation Medicine, Faculty of Medical Sciences, University of Fukui, 23-3 Matsuoka Shimoaizuki, Eiheiji-cho, Yoshida-gun, Fukui, 910-1193 Japan

**Keywords:** Outcomes research, Risk factors, Disability, Pain, Diseases, Neurological disorders

## Abstract

Osteoporosis and Parkinson’s disease (PD) are age-related diseases, and surgery for osteoporotic vertebral collapse (OVC) in PD patients become more common. OVC commonly affects the thoracolumbar spine, but low lumbar OVC is frequent in patients with lower bone mineral density (BMD). The aim of this study was to identify differences in clinical and imaging features of low lumbar OVC with or without PD and to discuss the appropriate treatment. The subjects were 43 patients with low lumbar OVC below L3 who were treated surgically, including 11 patients with PD. The main clinical symptoms were radicular pain in non-PD cases and a cauda equina sign in PD cases. Rapid progression and destructive changes of OVC were seen in patients with PD. The morphological features of OVC were flat-type in non-PD cases with old compression fracture, and destruction-type in PD cases without old compression fracture. Progression of PD was associated with decreased lumbar lordosis, lower lumbar BMD, and severe sarcopenia. High postoperative complication rates were associated with vertebral fragility and longer fusion surgery. Progression of postural instability as a natural course of PD may lead to mechanical stress and instrumentation failure. Invasive long-fusion surgery should be avoided for single low lumbar OVC.

## Introduction

The incidence of osteoporotic vertebral collapse (OVC) is likely to increase with aging of the population. Conservative management is effective in most cases, but OVC with back pain, neurological complications or a concern of paralysis requires surgery to permit a return to activities of daily life (ADL). In a multicenter cohort of 403 patients in Japan who underwent fusion surgery for OVC with neurological deficits, a thoracolumbar junction lesion at T12 and L1 was most frequent, but 18.9% of cases had low lumbar OVC below L3^1^. Clinical and imaging features in low lumbar OVC may differ from those in thoracolumbar OVC, based on our previous findings^2^.

Low lumbar OVC is also frequently found in patients with lower bone mineral density (BMD) and a higher mechanical failure rate, compared with thoracolumbar junction collapse^3^. Complications after surgery for OVC occurred in 14.1% of cases in a multicenter cohort study, with age, liver disease, and Parkinson’s disease (PD) found to be independent risk factors for postoperative complications^4^. In the same series, a comparison of patients with thoracolumbar spine (T10-L2) with and without PD found higher rates of perioperative complications and lower walking ability at follow-up in the PD group^5^.

PD is a common neurodegenerative disease with features of locomotor dysfunction, including resting tremors, rigidity, and bradykinesia. Patients with PD also often have low bone quality, loss of muscle mass (sarcopenia), and an abnormal posture. PD affects 1% of the population over age 60 and up to 4% over age 80, and the prevalence generally increases with age^6^. Thus, both OVC and PD are age-related diseases with low bone quality. Higher rates of postoperative complications and revision surgeries have been reported after spine surgery for patients with PD, such as fusion surgery for degenerative diseases and thoracolumbar OVC. However, clinical status, neurological symptoms, radiological findings, and surgical strategies for patients with low lumbar OVC with PD have not been described.

We hypothesized that the different clinical and imaging features in patients with low lumbar OVC with and without PD may be related to neurological symptoms. In this study, we examined these relationships, with the goal of proposing appropriate surgical treatments.

## Results

### Demographics of patients with and without Parkinson disease

Demographic data for patients who underwent surgery for low lumbar OVC below L3 are shown in Table 1. OVC occurred at L3 in 18 cases, L4 in 18, and L5 in 7. Of the 43 patients, 11 had PD, and 32 did not have PD (non-PD). There were no significant differences in gender, mean age at the time of surgery, history of other fragility fractures, comorbidities, steroid intake, medication for osteoporosis, affected vertebra levels, surgical procedures, and JOA scores before and after surgery in PD and non-PD patients. BMI was lower in PD cases, but with no significant difference to that in non-PD cases (19.7 ± 3.9 vs. 22.4 ± 3.7 kg/m^2^, *p* = 0.17). The mean follow-up period was 3.7 ± 1.0 (range 1–5) and 3.5 ± 2.0 (1–8.5) years in PD and non-PD cases, respectively. The Hoehn & Yahr stage before surgery was stage II in 5 patients and stage III in 6. Progression of severity of PD occurred in 4 of 11 patients (36.4%): 3 of 5 (60.0%) in stage II reached stage III, and 1 of 6 (16.7%) in stage III reached stage IV.

**Table 1:** Demographic data for patients with low lumbar osteoporotic vertebral collapse with and without Parkinson’s disease.

Item	PD patients	Non-PD patients	*p*
Number of patients	11	32	
Male	2	7	0.80
Female	9	25	
Age (years)	76.2 ± 4.1	76.4 ± 7.6	0.90
Hoehn & Yahr stage before surgery (at follow-up)	Stage II: 5 (2) Stage III: 6 (8) Stage IV: 0 (1)	–	
History of other fragility fractures	2	6	0.97
BMI (kg/m^2^)	19.7 ± 3.9	22.4 ± 3.7	0.17
**Comorbidities**			
Diabetes Mellitus	3	10	0.80
Rheumatoid arthritis	1	3	0.98
Steroid intake	1	4	0.76
Medication for osteoporosis before injury	3	16	0.19
Affected vertebra: number	L3: 6 L4: 4 L5: 1	L3: 12 L4: 14 L5: 6	0.72
Surgical procedures: number	VP: 1 VP + PSF: 5 APSF: 2 3CO: 1 LM: 2	VP: 4 VP + PSF: 9 PSF: 7 PLIF: 6 ASF: 2 APSF: 1 3CO: 1 LM: 2	0.11
Preoperative JOA score	11.8 ± 2.4	12.7 ± 3.7	0.52
Postoperative (2–4 weeks) JOA score	18.2 ± 2.4	18.7 ± 2.2	0.55
JOA score at follow-up	21.0 ± 2.4	22.1 ± 3.4	0.32
JOA improvement rate at follow-up	52.9 ± 16.0	58.2 ± 16.2	0.38
Follow-up period (years)	3.7 ± 1.0	3.5 ± 2.0	0.77

*PD* Parkinson disease, *BMI* Body mass index, *VP* Vertebroplasty, *PSF* Pedicle screw fixation, *PLIF* Posterior lumbar interbody fusion, *ASF* Anterior spinal fusion, *APSF* Anterior and posterior spinal fusion, *3CO* 3 Column osteotomy, *LM* Laminotomy.

### Clinical and imaging findings in patients with low lumbar OVC with and without PD

Symptoms and durations from diagnosis of compression fracture to that of burst fracture with neurological symptoms are summarized in Table 2. Most patients with and without PD complained of severe low back pain, but there was a significant difference in neurological symptoms between PD and non-PD cases. The main clinical symptom in non-PD cases was radicular leg pain (*p* = 0.016), whereas a cauda equina sign, such as motor and/or sensory disturbance, was most common in PD cases (*p* = 0.0047). Importantly, rapid progression of OVC was much clearer in PD cases compared with non-PD cases (24.5 ± 10.5 vs. 58.5 ± 33.4 days, *p* < 0.01).

**Table 2:** Differences in clinical symptoms and duration in patients with and without Parkinson’s disease.

Item	PD patients (n = 11)	Non-PD patients (n = 32)	*p*
**Symptom**			
Low back pain	10 (90.9%)	26 (81.3%)	0.45
Radicular pain	3 (27.3%)	22 (68.8%)	0.016*
Cauda equina sign	8 (72.7%)	8 (25.0%)	0.0047*
Duration (days)^†^	24.5 ± 10.5	58.5 ± 33.4	< 0.01*

^†^Duration from diagnosis of compression fracture to that of burst fracture and/or appearance of neurological symptoms.

*PD* Parkinson disease.

*p < 0.05.

Differences in imaging findings between patients with and without PD are shown in Table 3. On plain radiographs, non-PD cases were significantly more likely to have old compression fractures at the thoracolumbar level (1, 9.1% vs. 23, 71.9%, *p* = 0.0003). No patients had type 1 OVC (wedge-type), as often found in thoracolumbar OVC. There was a higher rate of cases with type 2 OVC (flat-type) in non-PD cases and of type 4 (destruction-type) in PD cases (*p* = 0.0073). Twelve of 23 type 2 cases (52.2%) had intervertebral cleft formation at the affected vertebra, but with no significant difference between the two groups. Preoperative lumbar BMD (*p* = 0.043) and L3 total psoas area/vertebral body area ratio (*p* = 0.044) were significantly lower in PD cases. There was no significant difference in preoperative lumbar lordosis and local lumbar lordosis before and after surgery, but PD cases had decreased lumbar lordosis at follow-up (*p* = 0.02).

**Table 3:** Differences in imaging findings in patients with and without Parkinson’s disease.

Item	PD patients (n = 11)	Non-PD patients (n = 32)	*p*
Presence of compression fracture at thoracolumbar level (%)	1 (9.1%)	23 (71.9%)	0.0003*
Type of osteoporotic vertebral collapse	Type 1: 0 Type 2: 3 (27.3%) Type 3: 2 (18.2%) Type 4: 6 (54.5%)	Type 1: 0 Type 2: 20 (62.5%) Type 3: 9 (28.1%) Type 4: 3 (9.4%)	0.0073*
Preoperative lumbar BMD (g/cm^2^)	0.58 ± 0.051	0.63 ± 0.080	0.043*
Appearance of intervertebral cleft (%)	3 (27.3%)	13 (40.6%)	0.43
Preoperative lumbar lordosis (degree)	15.8 ± 7.9	20.7 ± 12.0	0.24
Preoperative local lumbar lordosis (degree)	5.9 ± 11.6	− 2.2 ± 16.8	0.31
Lumbar lordosis at follow-up (degree)	7.7 ± 9.2	20.1 ± 13.5	0.02*
Local lumbar lordosis at follow-up (degree)	7.7 ± 10.6	7.8 ± 9.2	0.97
L3 total psoas area/vertebral body area (mm^2^)	0.45 ± 0.13	0.56 ± 0.20	0.044*

*PD* Parkinson disease, *BMD* Bone mineral density.

*p < 0.05.

### Postoperative complications and related factors

Postoperative complications occurred in 5 PD (45.5%) and 11 non-PD (34.4%) patients, including loosening and migration of pedicle screws (PS), progression of vertebral collapse, infection and delirium. In addition, exacerbation of dyskinesia was observed in one PD patient. The rate of revision surgery was higher in PD cases, but with no significant difference compared with non-PD cases (27.3% vs. 9.4%, *p* = 0.14) (Table 4). Data for postoperative complications related to instrumentation failures are shown in Table 5. Patients with postoperative complications had significantly lower preoperative lumbar BMD (*p* = 0.015). In addition, change of lumbar lordosis (*p* = 0.047), and longer fused vertebrae (*p* = 0.041) were also significant. No significant differences in BMI (*p* = 0.60), presence of PD (*p* = 0.51), type of OVC (*p* = 0.92), change of local lumbar lordosis (*p* = 0.84), L3 total psoas area/vertebral body area (*p* = 0.75), and surgical procedures (*p* = 0.87) were observed.

**Table 4:** Postoperative complications in patients with and without Parkinson’s disease.

Item	PD patients (n = 11)	Non-PD patients (n = 32)	*p*
Fused segments	3.3 ± 1.8	2.4 ± 1.4	0.26
Postoperative complications	5 (45.5%)	11 (34.4%)	0.51
Loosening and migration of pedicle screw (except for patients who underwent VP or LM)	4 (50.0%)	6 (23.1%)	0.14
Progression of vertebral collapse	3 (27.3%)	5 (15.6%)	0.39
Infection	1 (9.1%)	1 (3.1%)	0.42
Delirium	2 (18.2)	4 (12.5)	0.64
Exacerbation of dyskinesia	1 (9.1)	–	–
Revision surgery	3 (27.3%)	3 (9.4%)	0.14

*PD* Parkinson disease, *VP* Vertebroplasty, *LM* Laminotomy.

**Table 5:** Factors in postoperative complications related to instrumentation failures.

Item	With complications (n = 16)	Without complications (n = 27)	*p*
Age (years)	74.6 ± 7.9	77.3 ± 6.8	0.30
BMI (kg/m^2^)	22.3 ± 3.7	21.5 ± 4.0	0.60
With PD	5 (31.3%)	6 (22.2%)	0.51
Affected vertebra	L3: 8 L4: 6 L5: 2	L3: 10 L4: 12 L5: 5	0.76
Type of osteoporotic vertebral collapse	Type 2: 8 (50.0%) Type 3: 4 (25.0%) Type 4: 4 (25.0%)	Type 2: 15 (55.6%) Type 3: 7 (25.9%) Type 4: 5 (18.5%)	0.92
Preoperative lumbar BMD (g/cm^2^)	0.56 ± 0.067	0.65 ± 0.073	0.015*
Changes of lumbar lordosis (degree)	− 5.3 ± 9.6	0.86 ± 6.1	0.047*
Changes of local lumbar lordosis (degree)	3.6 ± 13.2	2.8 ± 8.2	0.84
L3 total psoas area/vertebral body area (mm^2^)	0.56 ± 0.17	0.54 ± 0.21	0.75
Surgical procedures	VP: 2 VP + PSF: 4 PSF: 4 PLIF: 3 APSF: 1 3CO: 1 LM: 1	VP: 3 VP + PSF: 9 PSF: 3 PLIF: 3 ASF: 2 APSF: 3 3CO: 1 LM: 3	0.87
Fused vertebra	3.1 ± 1.2	2.2 ± 1.5	0.040*

*BMI* Body mass index, *PD* Parkinson disease, *BMD* Bone mineral density, *VP* Vertebroplasty, *PSF* Pedicle screw fixation, *PLIF* Posterior lumbar interbody fusion, *ASF* Anterior spinal fusion, *APSF* Anterior and posterior spinal fusion, *3CO* 3 Column osteotomy, *LM* laminotomy.

*p < 0.05.

### Representative cases

**Figure 1:**
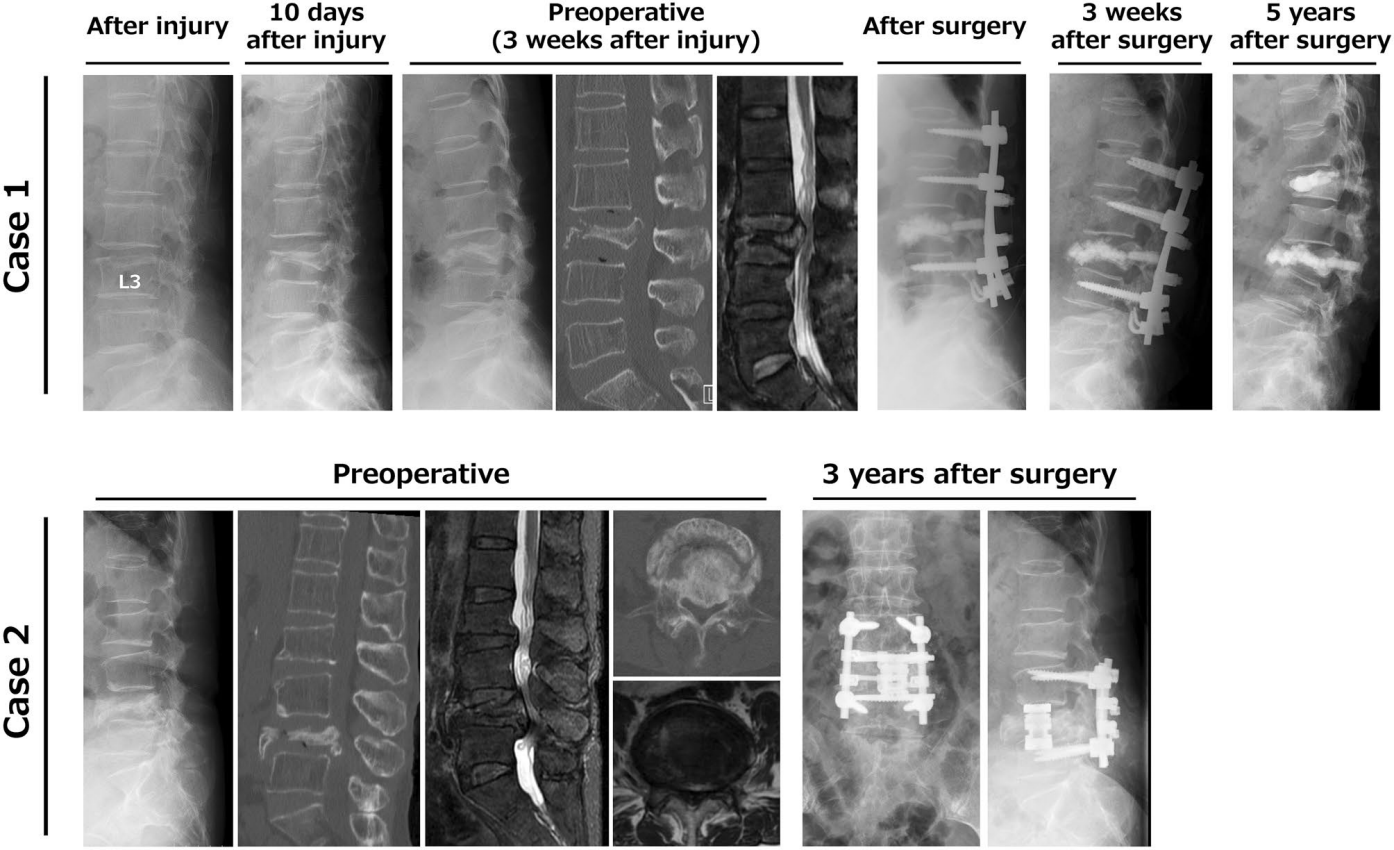
Case 1: A 74-year-old female (Hoehn & Yahr stage III) with severe low back pain and motor dysfunction due to rapidly progressive L3 osteoporotic collapse. Preoperative CT and T2-weighted MRI showed L3 collapse with canal stenosis by bony fragments that had retropulsed into the spinal canal. Instrumentation failure occurred early in the postoperative period with exacerbation of dyskinesia, and required removal of the pedicle screw, but daily activities were maintained despite a decrease in lumbar kyphosis at 5 years after surgery. Case 2: A 70-year-old female (Hoehn & Yahr stage II) with severe low back pain and motor dysfunction due to progressive L4 collapse. Preoperative CT and T2-weighted MRI showed L4 collapse with canal stenosis by bony fragments that had retropulsed into the spinal canal. The patient has been asymptomatic for 3 years after anterior reconstruction and pedicle screw fixation.

Pre- and postoperative images from two representative cases are shown in Fig. 1. Case 1 was a 74-year-old female (Hoehn & Yahr stage III) who presented with severe low back pain and a cauda equina sign (motor paralysis in both lower extremities) due to rapidly progressing L3 collapse within 3 weeks after injury. Vertebroplasty and PS fixation were performed, but PS loosening in upper segments occurred at 3 weeks after surgery with exacerbation of dyskinesia. The patient was able to maintain daily activities with only mild low back pain at 5 years after surgery, although decreasing lumbar kyphosis was observed after PS removal. Case 2 was a 70-year-old female (Hoehn & Yahr stage II) who presented with severe low back pain and a cauda equina sign (motor paralysis in both lower extremities) due to progressive L4 collapse. Anterior reconstruction and PS fixation were performed, and the patient has been asymptomatic for 3 years after surgery without instrumentation failure.

## Discussion

This study showed important differences in low lumbar OVC in patients with or without PD. Rapid progression and destructive changes of low lumbar OVC in patients with PD, and significantly more PD cases with a cauda equina sign requiring urgent surgery were important findings in this study. Fragility fractures are more likely to occur in patients with PD than in similarly aged non-PD subjects because of a higher risk of falls and lower BMD due to weight loss and lower mobility^7,8^. A meta-analysis showed an increased risk of fracture of 2 to 3 times in PD patients compared to controls^9^. A nationwide population-based study found a significantly increased risk of osteoporosis (hazard ratio 1.32) and surgery for OVC (hazard ratio 2.69) in PD patients compared to non-PD subjects^10^. Weight loss and sarcopenia are common in PD patients and correlate with greater motor changes, a higher rate of fall, and disease progression^11^. In a cross-sectional study, 55.8% of PD cases had sarcopenia compared to only 8.2% of controls^12^. There is also a strong negative correlation of BMD with the severity of PD, particularly for the lumbar spine^13,14^. Similarly, lower BMI, lumbar BMD, and sarcopenia were present in PD cases in the current study.

**Figure 2:**
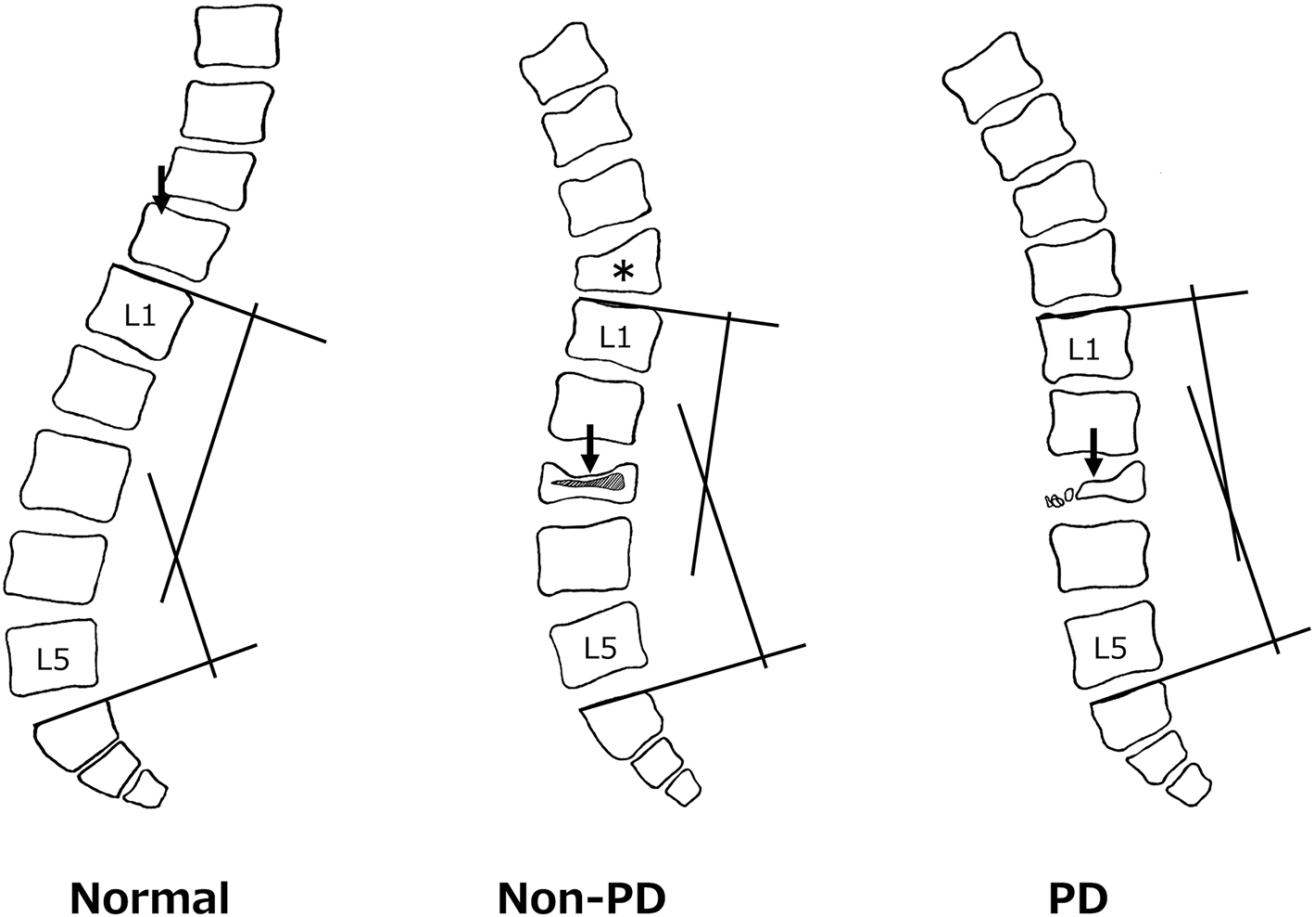
Differences in the pathomechanism of low lumbar osteoporotic vertebral collapse (OVC) in patients with or without Parkinson’s disease (PD). (**A**) OVC is most frequent at the thoracolumbar junction, such as at T12 and L1, due to anterior loading on the spine. (**B**) Most non-PD cases have old thoracolumbar compression and decreased lumbar lordosis (L1-S1). (**C**) Most patients with PD have decreased lumbar lordosis without old thoracolumbar compression. Cases with decreased lumbar lordosis (**B**, **C**) have increased middle and/or posterior loading on the low lumbar spine after changes in spinal alignment.

PD is a systemic and progressive disease, which suggests that the natural course of PD should be considered in treatment for OVC in a patient with PD. These aspects of PD might also influence clinical features and imaging findings in OVC. In the classification of OVC based on preoperative lateral radiographs, flat-type (type 2) low lumbar OVC was dominant in non-PD cases, whereas destructive-type (type 4) was found in most PD cases. The main clinical symptom in type 2 low lumbar OVC is radicular pain resulting from retropulsed bony fragments causing foraminal stenosis and/or canal stenosis^2^. In contrast, rapidly progressive OVC in type 4 could lead to cauda equina symptoms due to rapid and severe spinal canal stenosis. In addition, old compression fracture at the thoracolumbar level was found in most non-PD cases, but in few PD cases. Spinal alignment changes may increase middle and/or posterior low lumbar spine loading, and lower bone quality and severe sarcopenia in PD might cause rapid progression and destructive changes of OVC (Fig. 2).

We found relatively high rates of postoperative complications of 45.5% in PD cases and 34.4% in non-PD cases, mainly related to instrumentation. The rate of revision surgery was particularly high in PD cases (27.3%). In a large-scale cohort study of thoracolumbar spine fusion surgery using a national insurance database in the US, PD was significantly associated with an increased risk for medical complications (odds ratio 1.22) and revision surgery (odds ratio 1.70)^15^. Another large-scale matched-pair cohort study using an inpatient database in Japan also suggested that PD was a significant predictor of postoperative complications (odds ratio 1.74) following spine surgery, with delirium being most frequent^16^. In our study, postoperative complications were associated with lower preoperative lumbar BMD, decreased lumbar lordosis, and longer fusion surgery. In a multicenter study of lumbar spine surgery, surgical failure was more frequent in PD cases in fusion (45.8%) and corrective (67.7%) surgeries than in laminectomy only (33.3%). Thus, lower preoperative lumbar lordosis may be associated with failure of initial surgery, which suggests that use of rigid fixation to achieve and maintain proper lumbar lordosis may be effective in PD cases^17^. We agree with the necessity of long-segment corrective fusion surgery to maintain spinopelvic harmony for global sagittal malalignment, especially such as camptocormia in patients with a stooped posture due to PD progression. However, poorer outcomes and lower fusion rates in multi-level fusion surgery have also been reported for PD cases^18,19^, and PD patients treated with long instrumentation, surgery including the thoracic spine, and with less effective spinopelvic realignment tend to require earlier revision^20^.

In the present study, lumbar lordosis at follow-up was significantly lower in PD patients, although there was no significant difference in local lumbar lordosis at follow-up between PD and non-PD cases. A biomechanical study showed that a flexion loading condition increased stress by 39.5%-42.7% in the suprajacent disc^21^, and adjacent disc stress in longer fusion and fusions involving lower lumbar segments is higher than that in upper lumbar segments^22^. These results suggest that progression of postural instability in the natural course of PD could lead to mechanical stress and instrumentation failure, especially at an upper adjacent level. In the current study, the presence of PD alone was not correlated with instrumentation failure, but significantly lower lumbar BMD and decreased lumbar lordosis in PD patients might contribute to a higher rate of instrumentation failure compared to that in non-PD patients. An epidemiological study revealed camptocormia in 4.1% of PD patients^23^, and we found that sagittal spinopelvic alignment did not differ in low lumbar OVC cases with and without postoperative complications^2^. Long-fusion surgery should be avoided for patients with single-vertebra OVC because poor sagittal alignment is less common in such cases, but short fusion from a posterior approach cannot correct a spinopelvic mismatch and instrumentation should not be ended in a kyphotic segment.

The surgical indication and strategy should be considered based on the high possibility of deterioration of ADL in the natural course of PD, even if no postoperative complications occur. PD severity of stage III or above has been found to be a significant risk factor for further lumbar spine surgery^24^. In assessing long-term outcomes of PD patients, it should also be recognized that the rates of reaching stages III and IV by 5, 10, and 15 years after onset are 30.2% and 6.5%, 57.2% and 27.9%, and 83.5% and 41.2%, respectively^25^. In addition, the rates of dyskinesia, which increases the risk of instrumentation failure, at these time points are 8.4%, 35.1%, and 62.8%, respectively. Appearance of depression decreasing the motivation for rehabilitation after surgery and deterioration of ADL associated with progression of PD is also important in treatment of OVC. Depression can have a significant impact on severity of PD based on health-related quality of life^26^. In our study, 4 of 11 cases (36.4%) had progression of severity of PD at follow-up. Thus, medical management as well as surgical strategy are important to improve the outcomes of spinal surgery in patients with PD.

In surgery for type 2 or 3 low lumbar OVC in non-PD cases, short fusion from a posterior approach is ideal due to easier decompression for the lumbar canal and intervertebral foramen stenosis for fractured vertebra^2^. In PD cases, the surgical procedure is chosen based on severity of PD, type of OVC, neurological signs, endplate destruction, and kyphotic changes in the lumbar spine. For most patients with PD, additional reconstruction of the anterior column should be considered, especially for type 4 OVC. Anterior reconstruction using an expandable cage from a lateral lumbar approach and 1-above 1-below fixation as shown in case 2 in Fig. 1 might be ideal. In type 2 OVC in PD cases, VP + PS may be more likely to cause instrumentation failure and/or flesh compression fracture as shown case 1 in Fig. 1 than in non-PD cases, due to progression of postural abnormalities and decreased lumbar lordosis in the natural course of PD, which may lead to upper adjacent segment disease and degenerative spondylolisthesis. Because of rare PD cases with sclerotic changes such as in type 3 OVC, PS + PLF or PLIF are often not indicated. For a few patients with radicular pain or a high risk of instrumentation failure, there may be an option to wait for bone union with aggressive control of PD, and then perform laminectomy. These surgical strategies for low lumbar OVC in PD cases have been inferred from the pathological conditions observed in the present study and require verification in a future study.

This study has certain limitations. First, the study was retrospective, single-center design, and small number of patients without inclusion of cases receiving conservative treatment. Some results lack statistical power, and the results may change as the number of samples increases for some parameters that did not show a significant difference in the current study. Second, it had lack of use of minimally invasive surgeries, including anterior reconstruction and percutaneous pedicle screw fixation, which have been developed in recent years. In addition, the samples, including those examined in various types of surgery that may affect spinal alignment more than PD itself, might be too small for generalizability of the results to discuss surgical outcomes and strategies. Larger prospective and multicenter clinical studies are needed to provide further evidence in support of our results. Despite these limitations, we believe that our findings provide key insights on management of surgery for low lumbar OVC in patients with PD, since there are few reports on this condition in this patient population.

## Methods

### Study population

Between 2005 and 2018, 43 patients underwent surgeries for symptomatic low lumbar OVC at vertebral levels below L3 at our hospital and had a minimum follow-up period of 2 years. Eleven of these patients had PD, and clinical features and imaging findings in these patients were compared with those for non-PD cases (n = 32). No patients had comorbidities that caused lower extremity sensory or motor deficits directly, including symptomatic lumbar spinal canal stenosis. All patients underwent plain radiography, computed tomography (CT), high-resolution magnetic resonance imaging (MRI), and a BMD scan before surgery. Before surgery, written informed consent was obtained from each patient. The study protocol was approved by the Human Ethics Review Committee of Fukui University Medical Faculty (Approval Number 2014046) and strictly followed the Clinical Research Guidelines of the Ministry of Health, Labor, and Welfare of the Japanese Government.

### Outcomes and radiological measurements

Data for age, gender, body mass index (BMI), disease duration from diagnosis of compression fracture to that of burst fracture and/or appearance of neurological symptoms, surgical procedures, and postoperative complications were acquired from medical charts. In patients with PD, the Hoehn & Yahr stage before surgery and at follow-up was assessed by senior neurologists. Clinical symptoms, low back pain, radicular leg pain, and cauda equine sign were also stratified based on cleft formation. Radicular leg pain was defined as lower extremity pain consistent with a neurological dominant region. Cauda equina syndrome was diagnosed based on symptoms of neurogenic intermittent claudication or motor and/or sensory disturbance, including bladder dysfunction. The Japanese Orthopaedic Association (JOA) score for neurological status was obtained before surgery and at follow-up, with all neurological evaluations performed by senior spine surgeons. Neurological improvement rate at follow-up was calculated using the following formula: (postoperative follow-up JOA score—preoperative JOA score) × 100/(29-preoperative JOA score). Plain standing radiographs were used to identify other vertebral collapses, the type of vertebral collapse, lumbar lordosis and local lumbar lordosis before surgery and at follow-up.

**Figure 3:**
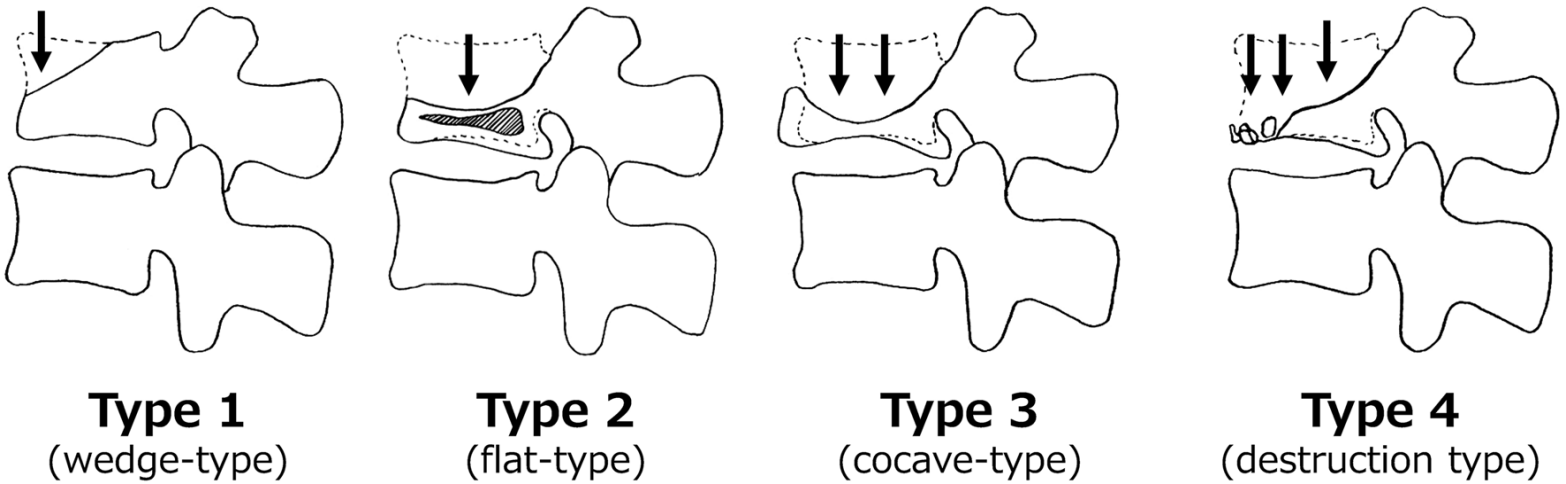
Osteoporotic vertebral collapse (OVC) is classified into four types based on findings on lateral radiographs and MRI: type 1 (wedge collapse), in which the ratio of anterior to posterior height of the vertebral body is < 60%; type 2 (flat or vertebra plana), with uniform compression and often intervertebral cleft formation; type 3 (concave), with an anterior spur or sclerotic changes, or an H shape; and type 4 (burst fracture), with severe destruction of the anterior vertebral body.

Each OVC was classified as one of four types based on its appearance on a lateral projection in a neutral position (Fig. 3)^2^: type 1 is a wedge-type collapse defined as a ratio of the anterior to posterior height of the vertebral body of < 60%; type 2 is a flat or vertebra plana-like fracture with uniform compression; type 3 is a concave or H-shaped fracture with anterior spur formation or sclerotic change; and type 4 is a burst fracture with severe destruction of the anterior vertebral body. Type 2 collapse often appears as intervertebral cleft formation on radiographs or MRI. Dual-energy X-ray absorptiometry (QDR 1000; Hologic) was used to measure mean BMD of the lumbar spine (L1-L4) in the posteroanterior projection excluding fractured vertebra. To assess the degree of sarcopenia, the sum of the left and right psoas area on the axial image in the middle of the L3 vertebral body divided by the area of the L3 vertebral body was calculated using a Picture Archiving and Communication System^27^. All measurements were performed in triplicate by each of two observers blinded to data related to surgery, and the average value was used.

### Surgical procedures

Vertebroplasty (VP) (n = 5) and a combination of decompression, VP, pedicle screw fixation, and posterolateral fusion (VP + PSF) (n = 13) were performed for cases with clefts at affected vertebra; whereas decompression, PSF, and posterolateral fusion (PSF) (n = 7), and posterior lumbar interbody fusion (PLIF) (n = 6) were used for cases without cleft formation. Other surgeries included correction osteotomy, including pedicle subtraction osteotomy (PSO) and vertebral column resection (VCR) (n = 2); anterior spinal fusion (ASF) using a plate system with iliac bone or a metal cage (n = 2); combined ASF with PSF (APSF) (n = 4); and laminotomy (LM) without fusion (n = 4).

### Statistical analysis

Data are shown as mean ± SD. Differences between groups were examined by Wilcoxon signed rank test, Mann–Whitney U-test or chi-square test, with *p* < 0.05 denoting significance. Inter- and intraobserver reliabilities were assessed using intraclass correlation coefficients (ICC). All calculations were performed using SPSS software (ver. 24.0, SPSS, Chicago, IL, USA). Power analysis were performed with EZR (Saitama Medical Center, Jichi Medical University, Saitama, Japan), which is a graphical user interface for R (The R foundation for Statistical Computing, Vienna, Austria).

### Ethics declarations

The study protocol was approved by the Human Ethics Review Committee of our University Medical Faculty (Approval Number 2014046) and strictly followed the Clinical Research Guidelines of the Ministry of Health, Labor, and Welfare of the Japanese Government.

## Data Availability

Data generated and analyzed during this study are included in this published article. Data and materials are available from the corresponding author subject to reasonable request and subject to the ethical approvals in place and materials transfer agreements.

## References

[1] Hosogane N (2019). Surgical treatment of osteoporotic vertebral fracture with neurological deficit: A nationwide multicenter study in Japan. Spine Surg. Relat. Res..

[2] Nakajima H (2016). Surgical treatment of low lumbar osteoporotic vertebral collapse: a single-institution experience. J. Neurosurg. Spine..

[3] Isogai N (2020). The surgical outcomes of spinal fusion for osteoporotic vertebral fractures in the lower lumbar spine with a neurological deficit. Spine Surg. Relat. Res..

[4] Sakai Y (2019). Complications after spinal fixation surgery for osteoporotic vertebral collapse with neurological deficits: Japan Association of Spine Surgeons with ambition multicenter study. J. Orthop. Sci..

[5] Watanabe K (2019). Surgical outcomes of spinal fusion for osteoporotic thoracolumbar vertebral fractures in patients with Parkinson’s disease: What is the impact of Parkinson’s disease on surgical outcome?. BMC Musculoskelet. Disord..

[6] de Lau LM, Breteler MM (2006). Epidemiology of Parkinson’s disease. Lancet Neurol..

[7] Malochet-Guinamand S, Durif F, Thomas T (2015). Parkinson’s disease: A risk factor for osteoporosis. Joint Bone Spine.

[8] Genever RW, Downes TW, Medcalf P (2005). Fracture rates in Parkinson’s disease compared with age- and gender-matched controls: a retrospective cohort study. Age Ageing.

[9] Tan L (2014). Parkinson’s disease and risk of fracture: a meta-analysis of prospective cohort studies. PLoS ONE.

[10] Lee CK (2018). Parkinson's disease and the risk of osteoporotic vertebral compression fracture: a nationwide population-based study. Osteoporos Int..

[11] Kim HJ (2012). Relationship between changes of body mass index (BMI) and cognitive decline in Parkinson’s disease (PD). Arch. Gerontol. Geriatr..

[12] Peball M (2019). Prevalence and associated factors of sarcopenia and frailty in Parkinson's disease: a cross-sectional study. Gerontology.

[13] Gao H (2015). Lower bone mineral density in patients with Parkinson's disease: a cross-sectional study from Chinese mainland. Front. Aging Neurosci..

[14] Zhao Y, Shen L, Ji HF (2013). Osteoporosis risk and bone mineral density levels in patients with Parkinson's disease: a meta-analysis. Bone.

[15] Puvanesarajah V (2016). Elective thoracolumbar spine fusion surgery in patients with Parkinson disease. World Neurosurg..

[16] Oichi T (2017). Mortality and morbidity after spinal surgery in patients with Parkinson’s disease: A retrospective matched-pair cohort study. Spine J..

[17] Kimura H (2017). Lumbar spinal surgery in patients with Parkinson disease: A multicenter retrospective study. Clin. Spine Surg..

[18] McClelland S (2017). Complications and operative spine fusion construct length in Parkinson’s disease: A nationwide population-based analysis. J. Clin. Neurosci..

[19] Bourghli A (2012). Posterior spinal fusion from T2 to the sacrum for the management of major deformities in patients with Parkinson disease: A retrospective review with analysis of complications. J. Spinal Disord. Tech..

[20] Sheu H, Liao JC, Lin YC (2019). The fate of thoracolumbar surgeries in patients with Parkinson’s disease, and analysis of risk factors for revision surgeries. BMC Musculoskelet. Disord..

[21] Srinivas GR, Kumar MN, Deb A (2017). Adjacent disc stress following floating lumbar spine fusion: a finite element study. Asian Spine J..

[22] Chen CS (2001). Stress analysis of the disc adjacent to interbody fusion in lumbar spine. Med. Eng. Phys..

[23] Seki M (2011). Keio Parkinson’s Disease Database. Camptocormia in Japanese patients with Parkinson’s disease: A multicenter study. Mov. Disord..

[24] Schroeder JE (2015). Lumbar spine surgery in patients with Parkinson disease. J. Bone Joint Surg. Am..

[25] Sato K (2006). Juntendo Parkinson Study Group. Prognosis of Parkinson’s disease: Time to stage III, IV, V, and to motor fluctuations. Mov. Disord..

[26] Global Parkinson’s Disease Survey (GPDS) Steering Committee. Factors impacting on quality of life in Parkinson’s disease: Results from an international survey. *Mov. Disord.* **17,** 60–67 (2002). 10.1002/mds.10010 11835440

[27] Bourassa-Moreau É (2020). Sarcopenia, but not frailty, predicts early mortality and adverse events after emergent surgery for metastatic disease of the spine. Spine J..

